# Factor structure and psychometric properties of the german version chronic uncertainty scale (CU-20)

**DOI:** 10.1186/s40359-023-01206-2

**Published:** 2023-05-30

**Authors:** Ileana Schmalbach, Christina Diane Bastianon, Walid A. Afifi, Gabriele Helga Franke, Andreas Hinz, Katja Petrowski

**Affiliations:** 1 Medical Psychology and Medical Sociology, University Medical Center of the Johannes Gutenberg University of Mainz, Duesbergweg 6 (Campus), 55128 Mainz, Germany; 2 Department of Communication, University of California Santa Barbara, Santa Barbara, CA USA; 3 Psychology of Rehabilitation, University of Applied Sciences Magdeburg and Stendal, Magdeburg, Germany; 4 Department of Medical Psychology and Medical Sociology, University of Leipzig, Leipzig, Germany; 5 Faculty of Medicine Carl Gustav Carus, Department of General, Technische Universität Dresden, Practice/MK3, Dresden, Germany

**Keywords:** Chronic uncertainty, Chronic stress, Factor analysis, Measurement invariance, CU-20, Psychometrics

## Abstract

**Background:**

The experience of uncertainty is ubiquitous and universal across the globe. Many available tools measuring uncertainty are focused on one aspect of uncertainty, e.g., patients with life-threatening illnesses, hence a measure considering (chronic) uncertainty as an integral experience reflect ongoing uncertainties from a socio-cultural perspective is missing. Additionally, current tools do not account for an extended timeframe to measure *chronic* forms of uncertainty. The objective of this study is to validate a translated German version of the 20 item Chronic Uncertainty Scale (CU-20).

**Methods:**

The full sample comprised N = 462 participants. Most of the participants were young German citizens and the sex distribution was relatively balanced (60% females; age in average: *M* = 24.56; SD = 4.78). Using equally split samples, an exploratory factor analysis (EFA) evaluated the CU-20 factor structure, followed by a confirmatory factor analysis (CFA) to test the established factor structure. Measurement invariance between male and female groups was evaluated. Internal consistency of the six-factor model was shown and scale discrimination was shown against chronic stress.

**Results:**

The EFA results showed decent model fit for the five-factor structure, however based on the CFA results, the theoretically established six-factor model fits the data significantly better. Measurement invariance between male and female groups was shown to be clearly scalar invariant. Cronbach’s alpha, omega and lambda all support internal consistency and reliability of CU-20.

**Conclusions:**

The CU-20 is a valid and reliable measure of one’s state of chronic uncertainty reflecting the individuals’ experiences of macrosocial forms of uncertainty, compared to the existing ones. This scale is especially useful in the context of migration, refugees or during global crises. Further psychometric testing is required in more diverse samples and a deeper look into measurement invariance is recommended.

## Background

Heraclitus’s realization that “nothing is more constant than change” [1] reframes well the general understanding of uncertainty. On one hand, reflecting a face of unpredictability and at the same time emphasizing the nature of living in a complex world. Reemphasizing this view, uncertainty surrounds many aspects of life [2] at different levels and not least as a result of a growing globalization in the sense of structural changes in contemporary societies, such as the breaking of traditions that used to structure individual choices (e.g., marriage, nuclear family, lifetime employment [3]). At an individual level, relationships (e.g., long-term commitments) may also pose a source of uncertainty [4]. At a macro level, immigration, financial crisis, pandemics (e.g., Covid-19 [5]), Ukraine war and climate change have a great potential in evoking feelings of uncertainty [4, 6]. For instance, as the COVID-19 virus spread civil unrest ignites national and global protests, and economic recessions looming, uncertainty has a revitalized meaning in the daily lives of people worldwide. According to Mishel’s [7] understanding, uncertainty is a cognitive state that occurs when one is unable to accurately predict outcomes, due to insufficient information, lack of control, unpredictability [8–10]. Further, it is perceived as a multidimensional [11] and dynamic state according to one’s perception of confidence and control [12], e.g., it can be perceived as an opportunity or as a threat [11, 13, 14]. Uncertainty is related with negative consequences for society at both levels, individual and globally. Firstly, uncertainty is known for being one of the major psychological stressors [15] associated with negative psychological (e. g., depression and anxiety; [16]) and health outcomes [17– 18], posing threat for well-being [13]. At a sociocultural level, extremism [4] and materialism [19] may arise as the consequence of living in conditions of increasing uncertainty, negatively affecting the immediate environment [20].

Further, the concept of uncertainty in the research at hand distinguishes from the construct of intolerance of uncertainty or fear of the unknown, which rather refer to intrapsychic experiences and conscious perception of absence of information [21–24] independently from the social context. In contrast, the manuscript at hand views uncertainty broadly speaking as the inability to know or predict outcomes [17, 25] and refers to a global phenomenon from a macro level viewpoint including socioeconomic, cultural and political aspects of uncertainty and draws on Warren and Ayton’s [26] phenomenological concept of uncertainty, referred as ‘the sense of uncertainty pervading everyday lived experience’. Therefore, the scale at hand aims to better reflect ongoing uncertainties in such domains, which can be chronic and generate individual experiences of chronic uncertainty. In this way, the scale would allow to tap individuals’ experiences of macro social forms of uncertainty, compared to the existing ones. Capturing and thus fostering understanding on individual experiences could enhance the effectiveness of psychological interventions by considering the context of exposure to adverse socioeconomic, cultural and political conditions [27] and counteract negative effects of uncertainty.

As uncertainty can take on a specific event focused, or a more general world view, the experience of uncertainty is universal across humanity [28]. As such, uncertainty has been studied in various realms of life including illness [29–31], migration [32], natural disasters [33, 34] and economic recession [35]. Hence, uncertainty illustrates the result of a state drawn from a very specific context. For example, in the case of health matters, only patients with chronic and/or life-threatening illnesses were questioned, resulting in uncertainty related to health-related matters, rather than (chronic) an integral experience of uncertainty. As a result, a more overarching measure of uncertainty is needed, especially since short-term and chronic uncertainty are ubiquitous phenomenon for many people (e.g., job uncertainty, diseases, financial burdens, social relationships).

Given the range of applications to which uncertainty is relevant, different measures of this construct are already in use. Considering the high interindividual response towards uncertainty, a valid screening tool is required. Hitherto, tools measuring uncertainty are specific to a given context, mostly in lieu of health/illness. For instance, the Mishel Uncertainty Illness Scale (MUIS) [35] and Uncertainty Stress Scale (USS; Hilton et al., [36] aiming to capture patient’s judgement of illness-related events. However, uncertainty is operationalized in two distinct ways, Mishel understands uncertainty is a ‘neutral cognitive state’ [37]; while Hilton depicts uncertainty as a cognitive perceptual state that can change over time and coexists with positive or negative feelings or emotions [36]. While both scales measure state constructs, this distinction foreshadows key discrepancies between the two scholars’ scales. Aside from uncertainty of illness, there are additional scales that focus on coping/response, e.g., the Uncertainty Response Scale (URS; Greco & Roger, [38]) or intolerance to uncertainty (IUS; Freestone et al., [39]). Furthermore, the Uncertainty Scale assesses patient symptom-based uncertainties that, unlike the Uncertainty in Illness Scale, are not necessarily linked to a “known disease state” [40]. Lastly, examples of target-specific scales beyond health contexts include the Relationship Uncertainty Scale [41], the Social Identity Uncertainty scale [42], and, an assessment of travel-related uncertainty [43]. Inherent to the concept of uncertainty, such may last for months or years, as currently experienced during the covid-19 pandemic or Ukraine war – since such situations are associated with lack of control and unpredictability. Hence, we aim to assess this self-report measure of chronic uncertainty, the Chronic Uncertainty (CU). Such is based on several studies revealing that experience of uncertainty is an ongoing and primary feature of life (e.g., Palestinian refugees).

To conclude, past evidence concentrates on uncertainty in patients with chronic and/or life-threatening illnesses, hence a measure considering (chronic) uncertainty as an integral experience in the general population is missing. This is remarkable, as short-term and chronic uncertainty are a ubiquitous in our society (e.g., job uncertainty, diseases, financial burdens, social relationships). As a result of this research gap, there is no available measurement, reference data or norm values on chronic uncertainty for the wider population. Therefore, this research investigated uncertainty with the aim to validate the newly translated German version of the 20 item Chronic Uncertainty Scale (CU-20) and provide reference group information and psychometric properties. This scale is being chosen as it aims to identify chronic forms of uncertainty on broader more general life aspects as compared to other scales which were developed with one specific population. The scale might be particularly useful in the context of immigration, refugees, financial crisis in order to better understand chronic uncertainty.

## Method

### Participants and procedure

The language used in this investigation was German and data were collected between summer 2019 and 2020 in a university in Germany (*N* = 473). We included participants between 18 and 65 years of age, no other criteria were required to participate. The surveys were implemented in two manners, via paper with data manually entered to EPI Data software v.4.6.0.2 [29] additionally, online via Sosci Survey [44]. In all cases, participants were informed of the study objectives and were given verbal and/or written information regarding data protection and their rights as participants; each participant gave voluntary consent in line with the Declaration of Helsinki. The study was approved by the ethic commission of Landesärztekammer Rheinland-Pfalz, Germany (2019–14,290).

Leading up to the current project, two stages of preliminary work have already been completed. First, translation and back-translation of the chronic uncertainty scale from English to German, originally and recently developed by Prof. Dr. Walid Afifi at the University of California, Santa Barbara (UCSB). Secondly, a pilot study using the German version of the chronic uncertainty scale was implemented in medical psychology and medical sociology (1st and 2nd semester) courses at Mainz University. The translation process of the CU-20 was in accordance with the International Test Commission (ITC) Guidelines for Translating and Adapting Tests [45]. Hence, the items were translated from German to English by one bilingual expert and then back-translated to German by a second bilingual expert. Comparison and reconciliation of the original and back-translated items was carried out by a group of experts, followed by a second round of forward and back-translation. The full sample comprised 60% females and a mean age of *M* = 24.56 (SD = 4.78, range 18–76). Only 9% of the sample were of a nationality other than German. The majority ascribed to Protestant (28%) or Catholic (36%) religious views. Additionally, 50% of participants reported being in a long-term partnership without being married, 38% were not in partnership, 5% were changing partners, and 6% were married.  

### Psychological measures

*Chronic uncertainty* scale (CU; Afifi & Afifi) [15]. The Chronic Uncertainty scale was originally developed from data collected in several chronic-uncertainties contexts, including Palestinian refugee camps in Lebanon [46, 47], undocumented immigrant communities in the United States [48], hardships following the 2008 recession [25], and natural disasters [33], combined with a review of literature on illness uncertainty [35] and on the experience of communities struggling with chronically-uncertain environments [49], among others. The result was the articulation of six domains of threat-related uncertainty: (1) *Safety*, (2) *Finances*, (3) *Relational*, (4) *Country*, (5) *Health*, and (6) *Separation from family*. The scale originally included 40 items but, with participant fatigue in mind, and with the goal of making the scale more accessible to community-based research, it has been reduced to 20 items, with each domain assessed through 2 to 4 items. Participants rated each item on the following 6-point scale: “Extremely Uncertain”, “Mostly Uncertain”, “Somewhat Uncertain”, “Somewhat Certain”, “Mostly Certain”, and “Extremely Certain”. Importantly, the scale was developed with the express function of being able to add or eliminate domains according to the particulars of the community and context in question. More specifically, while safety, health, and finances are universal aspects of life around which every individual can assess levels of uncertainty, experiences tied to relational well-being, concerns with the conditions within the country of residence or the country with which they identify as residents, and the potential for separation from family are more context-specific.

*Trier inventory for chronic stress* (TICS [50, 52]). The TICS is a validated scale that measures nine interrelated factors of chronic stress including *Work Overload, Social Overload, Pressure to Perform, Work Discontent, Excessive Demands from Work, Lack of Social Recognition, Social Tensions, Social Isolation* and *Chronic Worrying* [50, 51, 70, 71]. The original 57-item was reduced to a short 9-item version where each item represents one of the original nine aspects of chronic stress [35]. Response options are “Never”, “Rarely”, “Sometimes”, “Frequently”, and “Always”. The sum ranges between 9 and 45 where a higher overall score indicates higher chronic stress. The single factor structure is valid, highly correlated to the full-item scale (*r* = .91), high reliability via McDonald’s omega of 0.88, and shows measurement invariance between males and females as well as age groups [35].

### Statistical analyses

All statistical analysis were conducted using R version 3.6.3 and associated packages: lavaan [53], semPlot [54], semTools [55], psych [56], mice [57], GPArotiation [58], and tidyverse [59]. The total sample included *N* = 474 participants. Observations with greater than 15% missing data were dropped (n = 24), of which 14 had complete missing data, 10 had between 20 and 55% missing. Multiple imputation based on 25 iterations was used to complete the remaining observations missing 15% data or less (n = 40). Item characteristics including mean, standard deviation, item-difficulty, and corrected item-total correlations were evaluated (see Table 1 for all item-specific statistics). Normality was checked by evaluating item skewness, kurtosis and Shapiro-Wilks test. In the following, the statiscal analyses on the validity and reliability are described.

### Construct validity

Since conducting both the exploratory factor analyses (EFA) and the confirmatory factor analyses (CFA) using the same sample would lead to artificially increased model fit values, the total sample was randomly split into two equally sized sub-samples of 225 (χ²(1) = 1.12, *p* = .289, *V* = 0.053; Males: Sample 1 = 36,88%; Sample 2 = 42,22%. Females: Sample 1 = 63,11%; Sample 2 = 57,77%. Theoretical background suggested a 6-factor solution and a parallel analysis suggested a 5-factor solution guided further factor identification. An EFA using oblique rotation and maximum likelihood method (ML) was conducted to test the five-factor solution. Acceptable eigenvalues are 0.4 and higher, items with eigenvalues less than 0.4 and cross loading were considered for removal [60]. The assumption of sampling adequacy was examined via Bartlett’s test of correlation and the Kaiser-Meyer-Olkin test of sample adequacy.

Confirmatory factor analysis tested the theoretical 6-factor model and the EFA established 5-factor models in the second half of the original sample (*N* = 225). Due to data non-normality, robust and Satorra-Bentler corrections were used. Both CFAs were computed using robust maximum likelihood method and the variance of each factor was set to 1 for scaling. Satorra-Bentler corrected model fit indices were evaluated and compared to determine best model fit. The chi-square statistic (χ²), comparative fit-index (CFI), Tucker-Lewis Index (TLI), standardized root mean square residual (SRMR), root mean square error of approximation (RMSEA) and its 90% confidence interval (90% CI) were evaluated to determine model fit. Good model fit [adequate model fit] are indicated by CFI and TLI higher than 0.95 [> 0.90], SRMR below 0.05 [> 0.10], RMSEA values below 0.05 [< 0.08] [61, 62].

Measurement invariance across male and female groups was tested using the same CFA sub-sample (*N* = 225). Following recommendations of Milfont et al. [63], the theoretical six-factor model was tested in four increasingly constrained models: (1) configural, (2) metric, (3) scalar, and (4) strict. Model (1) tested that the CU-20 six-factor structure is invariant in both male and female groups. Model (2) held factor loadings equal across groups, followed by model (3) that additionally constrained item-intercepts. Lastly, model (4) constrained factor loadings, intercepts and error variances between male and female groups. Measurement invariance was evaluated by changes (Δ) in goodness of fit indices including: Δχ^2^, ΔCFI, ΔTLI, ΔSRMR and ΔRSMEA. When sample sizes are unequal, as is the case in this study, Chen [64] and Milfont [63] recommend the following cutoff criteria [adequate cutoff] for testing levels of invariance: a decrease of CFI (ΔCFI) by less than or equal to 0.005 [0.01] in magnitude, ΔRSMEA ≤ 0.010 [0.015] or a ΔSRMR ≤ 0.025 [0.30] (≤ 0.005 [0.10] for intercept and residual invariance) indicate invariance.

Scale discrimination was tested to further evaluate the CU construct validity. Pearson’s pairwise correlations of scale and sub-scale scores between the chronic uncertainty scale and the Trier Inventory of Chronic Stress are presented.

### Reliability

Internal consistency for each factor was tested with two related samples. For the full sample (n = 450) Guttman’s Lambda (λ) [65], Cronbach’s alpha (α), and McDonald’s omega(ω) were calculated, with 0.7 adopted as a floor for acceptable reliability [66, 67]. In connection with the CFA analysis (n = 225), Cronbach’s alpha and McDonald’s omega [66] were calculated.

## Results

### Item descriptive statistics

The descriptive item statistics for the full sample are presented in Table 1. Additionally presented are corrected item-test correlations and factor reliability measures of Cronbach’s a and Guttman’s *λ*. Shapiro-Wilks test of normality for all 20 items W > 0.67 (*p* < .001) resulted in significant non-normality.

Table 1. illustrates that the estimated alpha without the specific item (α_item_) identifies two items (5 and 12) that do not influence the scale alpha score. The corrected item-test correlation for all items is greater than 0.5 suggesting good item discrimination.

Table 2. shows the pearson pairwise correlations indicating that three item pairs correlated with a 0.90 or higher with correlations among the remaining item pairs ranging between 0.37 and 0.89.

### Construct validity

EFA: Bartlett’s test of sphericity on the EFA sample showed acceptable intercorrelations (χ²(190) = 5346, *p* < .01). As additional evidence for scale factorability, Kaiser-Meyer-Olkin Measure of Sampling Adequacy (KMO = 0.94) was greater than the 0.7 suggested cut-off for measuring the shared variance between item-pairs and no single item had a value lower than 0.89. The parallel analysis suggested a five-factor solution that is greater than chance.

An EFA using oblimin rotation and maximum likelihood method tested the 5-factor model that the parallel analysis recommended. Accepted item loadings ranged from 0.43 to 0.97. Only *Finances* and *Relational* factors maintained the items from the original English version; the *Safety* and *Security* factors merged together. Other items either loaded on multiple factors or had a primary loading on a factor different than its original theoretic placement. Item 1 is complex and loaded on the merged factor (0.47) along with the theoretically proposed *Health* factor (0.39). Item 18 loaded to the *Country* factor rather than the merged *safety-separated* factor and Item 17 is complex with loadings less than 0.40 on *Country* (0.38) and the merged *Safety-Separated* (0.35) factor.

CFA: Using the second half of the original dataset, two robust maximum likelihood method CFAs modeling the theoretical 6-factor structure from the English version and the EFA established 5-factor structure were conducted.

**Fig. 1:**
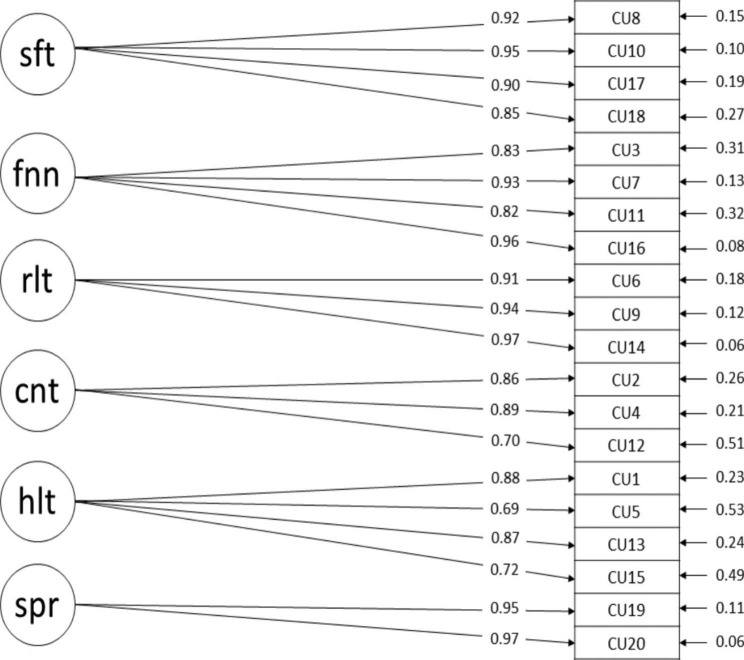
Chronic Uncertainty Scale theoretical 6-factor structure Note: sft = Safety, fnn = Financial, rlt = Relational, cnt = Country, hlt = Health, spr = Separated, covariances between latent variables range between 0.595 and 0.969

Figure 1. shows the 6-factor model and Table 3. presents the robust and Satorra-Bentler corrected CFA goodness of fit indices. The originally proposed 6-factor model showed good fit with the data. The CFI meets the 0.95 cutoff while the TLI was adequate. RMSEA and its 90% CI were within an acceptable range and below the adequate 0.08 cutoff. Additionally, SRMR was well below the recommended 0.05 cutoff, however as expected the χ² was significant. The high covariance between the *Safety* and *Security* factors signals concern regarding proper factor structure. The remaining covariances between factors were moderate to high ranging between. Items 5 and 12 were the weakest items in relation to their latent variable, as both had higher standard errors and lowest R^2^ estimates respectively.

The 5-factor model fit indices did not show evidence of good fit: The χ² was significant (χ²(100) = 333.9, p < .01) and RMSEA is above the adequate 0.08 cutoff (90% CI ranges from 0.09 to 0.114). Moreover, although CFI (0.95) met the 0.95 cutoff, TLI (0.91) was only adequate > 0.90. Table 3. shows the fit indices of the 5-factor model. Similar to the 6-factor model, the CFI and TLI surpass the cutoff supporting good model fit. RMSEA and its 90% CI were less than 0.08 and SRMR was below the 0.10 cutoff tending towards adequate model fit. Again, as expected χ² was significant, signifying poor model fit. As the *Separated* and *Safety* factors basically merged together, the factor covariances were no longer exceedingly high, rather they range between 0.66 and 0.86. As in the 6-factor model, items 5 and 12 continued to be the weakest items in relation to their respective factors, they had higher standard errors and R^2^ for both items were clearly lower than other items.

In sum, when comparing the fit indices in the 6- and 5-factor similar values can be observed, with the exception that SRMR is significantly better in the 6-factor solution (see Table 3). Therefore, in combination with theoretical support and primary motives of the scale the 6-factor model was favored and continued with measurement invariance testing.

### Measurement invariance

The established 6-factor CU scale was tested for measurement invariance between males and females using robust maximum likelihood method. The robust and Satorra-Bentler corrected model fit indices for each of the four hierarchical models are presented in Table 3. In evaluating measurement invariance, the change in goodness of fit of the four models is the focus and can be seen in Table 3. Based on the recommendations of Chen [64], the ΔCFI, ΔSRMR and ΔRMSEA for the metric and scalar models clearly indicate measurement invariance at the scalar level. With respect to the strict model, the ΔSRMR and ΔRMSEA support measurement invariance, however the ΔCFI just slightly exceeds the less-conservative cutoff of 0.10. Therefore, it could be argued that the CU-20 scale tends towards strict invariance between males and females, as pointed out by the configural invariance test. In sum, the CU-20 six-factor structure is invariant in both male and female groups. Metric invariance held factor loadings equal across groups, scalar invariance test additionally constrained item-intercepts. Lastly, strict invariance constrained factor loadings, intercepts and error variances between male and female groups.

In order to determine scale discrimination, we correlated the CU scale and sub-scales with the TICS total-scale.

Table 4. illustrates low – moderate Pearson’s pairwise correlations coefficients between the TICS and CU-Subscales, confirming discrimination validity.

### Reliability

Internal consistency was tested on the same sub-sample as the CFAs (*N* = 225).

As shown in Table 5., Cronbach’s alpha [67], McDonald’s omega [66] are strong and therefore support the CU-20 scale reliability and internal consistency.

## Discussion

Reemphasizing Heraclitus’s view that “nothing is more constant than change” [1], uncertainty is ubiquitous and might threaten psychological health and well-being if it becomes chronic and cannot be coped with efficiently [68, 69]. Past evidence on said construct has been mostly illness-related and measured in very specific contexts. Chronic and general uncertainty has not been vastly examined yet. Therefore, the current study examined the psychometric properties of the Chronic Uncertainty scale (CU-20). In addition, we evaluated the 6-factor scale for measurement invariance in gender.

Results from this study present a promising start for the CU-20 scale development. The 6-factor model fit indices were good, providing evidence for construct validity. Additionally, the scales’ high internal consistency supports scale reliability, which is in line with past research [17]. However, there were two points of concern regarding the 6-factor structure, in particular items 5 (“Getting through the day without physical struggle.”) and 12 (”The country’s commitment to protect all of its citizens.”) tend to be weakly related to their latent variables and do not contribute much explained variance in the model. These items stem from the original model that was intended to measure uncertainty in refugee camps or at natural disasters (Afifi et al., [65]). Item 12 seems to relate to political and social danger, as is the case for refugees or in the aftermath of a natural disaster, and perhaps less relevant our sample. Our sample comprises young German citizens with at rather stable life-style. However, given a general German population during the COVID-19 pandemic, item 12 may as easily relate to the government’s efforts to develop and implement lockdown measures and vaccination protocols. Therefore, the scale can be used generally but also items and the context can be slightly adapted to a given population or situation.

Further, the 6- and 5-factor displayed similar values. However, the SRMR-Index was significantly better in the 6-factor solution. Besides the latter and in combination with theoretical support and primary motives of the scale, we favored the 6-factor solution. Considering that a major aim of the scale is to capture broader and more general life aspects as compared to scales with specific populations merging *safety* and *separated* would contradict the pursued aims. Hence, it makes sense to differentiate between the factors *safety* and *separated*, especially considering that the development of the scale was based on the experience of refugees it makes sense to look at these life domains to capture the individual experience in a given surrounding. Moreover, a differentiated analysis is in the context of refugees or immigration important for diagnostic purposes, since it can be the case that refugees are separated from their families but safe. Lastly, some countries or contexts are more prone to higher levels of uncertainty and such distinctions are crucial for cross-cultural comparisons. Next, the covariance between the *safety* and *separated* factors are exceptionally high, suggesting that these factors may need additional adjustment with further testing. However, the results of the 5-factor model presented contradicting evidence as the two factors do not completely merge together, rather items 1 (“Being healthy enough to do daily activities.“) and 18 (“Your safety in your neighborhood.“) load on unexpected factors and item 17 (“Feeling secure in your neighborhood.“) does not properly load on any factor. Therefore, while the 6-factor model shows high covariance between the *safety* and *separated* factors, a 5-factor model is an unsatisfactory solution. Naturally, due to the non-normality of the data and moderate sample size, continued testing is warranted.

The 6-factor CU-20 was tested for measurement invariance between males and females and the results clearly showed scalar invariance. Scalar invariance between males and females allows for the interpretation of the scale means between groups. As the male and female samples differ in size the χ² may be biased [64]. Pearson’s pairwise correlation coefficients were, as expected, not particularly high, representing the minor relationship between chronic stress and chronic uncertainty. While stress and uncertainty are related concepts, they are by no means synonymous of one another and these results further support this point. To determine discriminant validity, we correlated the construct of chronic uncertainty (CU-20) and chronic stress (TICS) in order to determine to which degree our measure of target (CU-20) diverges or does not correlate with chronic stress. Our data provides evidence for discriminant validity since CU-20 positively correlates with TICS, but only low - moderate. This confirms the discriminant validity sought in the manuscript at hand. While TICS measures chronic stress focusing on unmet needs (e.g., appreciation, social support), CU-20 emphasizes cognitive features operationalized in its items. Nevertheless, our results are preliminary and further studies are needed in order to further confirm the discriminant validity.

To the best of our knowledge, this is the first study evaluating the psychometric properties of the CU-20 in a German population, however there are several limitations to consider. One limitation refers to the non-normality of the data which can reduce the power of the analysis. The data collected span across two years, which may introduce a bias through uncontrolled historical situations of participants’ socioeconomic or political perspectives of uncertainty. The sample was comprised of relatively young students from one university in Germany, thus the variation in scale responses were more homogenous and consistent. This and the non-normality of the data may have hindered the strength of analyses. While the sample size for the EFA and CFA analyses met the recommended size to conduct such analyses, the measurement invariance analysis suffered from slightly unequal and small sample size for each group (i.e., descriptively more females than males participated). Therefore, the measurement invariance model fit indices may be biased as a result [63, 64]. Lastly, scale discrimination related chronic stress is limited. While the 9-item TICS scale measures a single factor general chronic stress, the area-specific factors such as *Work Overload*, *Social Overload* and *Chronic Worrying* are not individual factors and therefore cannot be individually evaluated; furthermore, the original 57-item scale was developed in a German working context [50, 51]. This is in contrast to the CU scale development in the context of refugees and natural-disaster aftermath, where broader and more life-essential aspects are the focus.

Future research will need to continue testing the theoretically established 6-factor model specifically in larger and more diverse samples that more closely represent the German population. Refugees living in German refugee camps, first- and second-generation migrants, patients with chronic medical conditions, Germans with low socioeconomic status and the general German population in the context of Covid-19 provide example populations to further test the validity of the CU-20. The scale should also be tested within a concise timeframe to counter any potential bias from historical situations. However, a properly designed longitudinal study that accounts for changes over time would be valuable to develop the chronic aspect of the scale. Convergent validity of the German CU-20 should be evaluated. As there are no known German scales measuring uncertainty, measures for ambiguity or worry may provide limited insight, however a distinction should be made to trait measures such as the Intolerance to Uncertainty scale [39, 76], which measure a fundamentally different construct where one’s negative beliefs towards uncertainty is captured. Discriminant validity should continue to be tested particularly against acute and chronic stress measures. As concepts, uncertainty, stress, anxiety, and control have complex theoretical associations that are too often conflated and require additional scrutiny [49, 59], among others, for efforts to untangle. Additional developments in theoretical frameworks regarding uncertainty and stress will prove helpful across academic disciplines.

## Conclusion

The study at hand tested the factor structure, psychometric properties, and measurement invariance of the newly translated German version of the Chronic Uncertainty Scale. The six-factor structure is valid and the factors show strong reliability. The scale clearly shows scalar measurement invariance between males and females. The results are promising as we found evidence for construct and discriminant validity. This scale might provide a better understand of the concept of uncertainty at an individual level considering daily life circumstances, including crises, global events (e.g., war, pandemics) and can be of special use in the context of immigration, relocation, refugees. Further results may expand theoretical and practical implications for psychologists and public health domains.

**Table 1:** Item characteristics of the German version CU-20

	*item*	*mean*	*SD*	*median*	*S*	*K*	*se*	*α*	*α*_*item*_	*λ*	*r*_*it*_	*difficulty*
Safety	cu8	2.81	1.97	2	0.59	-1.3	0.09	0.95	0.94	0.95	0.87	0.47
	cu10	2.66	2.00	2	0.74	-1.16	0.09		0.93	0.95	0.89	0.44
	cu17	2.88	1.78	2	0.55	-1.12	0.08		0.92	0.95	0.91	0.48
	cu18	2.86	1.67	2	0.57	-0.95	0.08		0.94	0.95	0.86	0.48
Finances	cu3	3.08	1.71	3	0.35	-1.19	0.08	0.94	0.93	0.93	0.83	0.51
	cu7	2.95	1.82	2	0.5	-1.24	0.09		0.91	0.93	0.9	0.49
	cu11	3.04	1.78	3	0.36	-1.28	0.08		0.93	0.93	0.83	0.51
	cu16	2.88	1.84	2	0.53	-1.23	0.09		0.92	0.93	0.89	0.48
Relational	cu6	3.12	1.78	3	0.34	-1.31	0.08	0.96	0.95	0.94	0.89	0.52
	cu9	2.92	1.81	2	0.46	-1.25	0.09		0.93	0.94	0.91	0.49
	cu14	2.97	1.8	2	0.42	-1.26	0.08		0.92	0.94	0.93	0.50
Country	cu2	3.32	1.49	3	0.18	-1.14	0.07	0.87	0.77	0.83	0.81	0.55
	cu4	3.08	1.54	3	0.4	-1	0.07		0.82	0.83	0.76	0.51
	cu12	3.26	1.5	3	0.17	-1	0.07		0.87	0.83	0.71	0.54
Health	cu1	2.76	1.84	2	0.68	-1.08	0.09		0.83	0.87	0.76	0.46
	cu5	3.02	1.53	3	0.41	-0.93	0.07	0.87	0.87	0.87	0.65	0.50
	cu13	2.75	1.7	2	0.62	-0.94	0.08		0.80	0.87	0.81	0.46
	cu15	2.85	1.4	3	0.54	-0.63	0.07		0.85	0.87	0.72	0.47
Separated	cu19	2.62	2.12	1	0.76	-1.24	0.10	0.96	0.92	0.92	0.92	0.44
	cu20	2.61	2.16	1	0.79	-1.24	0.10		0.85	0.92	0.92	0.44

Note: for all items sample size N = 450, minimum = 1, maximum = 6. S = skew. K = kurtosis α = Cronbach’s α. α item = alpha minus the item

λ = Guttman’s λ. r_it_ = corrected item-total correlation

**Table 2:** Item pairwise correlations

		1	2	3	4	5	6	7	8	9	10	11	12	13	14	15	16	17	18	19	20
cu1	^H^	1																			
cu2	^C^	0.53	1																		
cu3	^F^	0.57	0.51	1																	
cu4	^F^	0.57	0.76	0.55	1																
cu5	^H^	0.65	0.45	0.45	0.5	1															
cu6	^R^	0.58	0.43	0.49	0.48	0.47	1														
cu7	^F^	0.66	0.49	0.8	0.58	0.53	0.61	1													
cu8	^S^	0.74	0.6	0.6	0.67	0.56	0.55	0.72	1												
cu9	^R^	0.64	0.47	0.52	0.52	0.5	0.86	0.64	0.61	1											
cu10	^S^	0.77	0.64	0.65	0.7	0.58	0.58	0.74	0.87	0.66	1										
cu11	^F^	0.61	0.51	0.74	0.57	0.52	0.52	0.8	0.68	0.55	0.71	1									
cu12	^C^	0.42	0.7	0.42	0.63	0.39	0.37	0.45	0.51	0.42	0.53	0.45	1								
cu13	^H^	0.73	0.47	0.56	0.52	0.55	0.57	0.63	0.66	0.62	0.68	0.57	0.43	1							
cu14	^R^	0.62	0.46	0.49	0.51	0.47	0.88	0.62	0.61	**0.91**	0.65	0.55	0.42	0.64	1						
cu15	^H^	0.58	0.34	0.45	0.4	0.52	0.48	0.51	0.47	0.49	0.48	0.45	0.35	0.8	0.51	1					
cu16	^F^	0.7	0.53	0.78	0.59	0.54	0.59	0.89	0.75	0.64	0.8	0.79	0.48	0.63	0.65	0.47	1				
cu17	^S^	0.71	0.64	0.63	0.66	0.56	0.54	0.7	0.82	0.63	0.83	0.68	0.55	0.67	0.6	0.52	0.74	1			
cu18	^S^	0.66	0.63	0.59	0.64	0.52	0.49	0.65	0.76	0.6	0.79	0.64	0.54	0.61	0.56	0.46	0.69	**0.9**	1		
cu19	^Se^	0.78	0.6	0.61	0.69	0.6	0.62	0.73	0.85	0.68	0.88	0.66	0.5	0.69	0.66	0.48	0.78	0.81	0.75	1	
cu20	^Se^	0.8	0.59	0.64	0.66	0.59	0.59	0.74	0.85	0.66	0.88	0.67	0.48	0.7	0.65	0.51	0.78	0.79	0.75	**0.92**	1

Note: Pearson correlations. correlations in bold ≥ 0.90

**Table 3:** Confirmatory Factor Analysis and Measurement Invariance Tests

	χ²	Δχ²	df	Δdf	p-val.	CFI	ΔCFI	TLI	ΔTLI	SRMR	ΔSRMR	RMSEA	ΔRMSEA	RMSEA lower	90% CI upper
CFA 5-Factor	293.923		142		< 0.01	0.958		0.949		0.071		0.08		0.067	0.093
CFA 6-Factor	327.52		155		< 0.01	0.954		0.943		0.037		0.084		0.071	0.097
Measurement invariance															
Configural	529.301		310		< 0.01	0.946		0.934		0.048		0.093		0.079	0.106
Metric	562.796	33.495	324	14	< 0.01	0.942	-0.004	0.932	-0.002	0.064	0.016	0.094	0.001	0.081	0.107
Scalar	586.206	23.41	338	14	< 0.01	0.941	-0.001	0.934	0.002	0.065	0.001	0.093	-0.001	0.08	0.105
Strict	643.163	56.957	358	20	< 0.01	0.93	-0.011	0.926	-0.008	0.064	-0.001	0.098	0.005	0.086	0.11

Note: Bentler scaled χ², p-val. Robust estimates for CFI, TLI, RMSEA and confidence interval (CI). Δ: difference of the model from the model before (metric = metric-configural)

**Table 4:** Pearson correlations of scale scores

	1	2	3	4	5	6	7	8
1 cu.safety	1							
2 cu.relational	0.66	1						
3 cu.finances	0.8	0.65	1					
4 cu.country	0.73	0.53	0.62	1				
5 cu.health	0.77	0.68	0.71	0.59	1			
6 cu.sep	0.9	0.68	0.78	0.67	0.78	1		
7 cu.score	0.94	0.8	0.89	0.78	0.87	0.92	1	
8 tics.score	0.22	0.26	0.25	0.26	0.25	0.27	0.28	1

Note: pairwise complete observations. cu.sep = separation sub-scale

**Table 5:** CU scale internal consistencies

6-factor				5-factor			
	α	ω	avevar		α	ω	avevar
Safety	0.950	0.945	0.832		0.965	0.9693	0.858
Finances	0.938	0.934	0.795		0.938	0.9344	0.795
Relational	0.957	0.957	0.882		0.957	0.9567	0.882
Country	0.857	0.855	0.672		0.857	0.8696	0.617
Health	0.869	0.875	0.655		0.819	0.8613	0.684
Separated	0.955	0.956	0.916				
Total	0.970	0.989	0.799				

Note: N = 225 uses the same sample as the CFA analysis. α = Cronbach’s alpha, ω = McDonald’s omega, Avevar = average extracted variance

## Data Availability

The datasets used and/or analyzed during the current study are available from Prof. Katja Petrowski: kpetrows@uni-mainz.de on reasonable request.

## Abbreviations

COVID-19: Corona virus
MUIS: Mishel Uncertainty in Illness Scale
USS: Uncertainty Stress Scale
URS: Uncertainty Response Scale
GAD: Generalized Anxiety Disorder
IUS: Intolerance to Uncertainty Scale
CU: Chronic Uncertainty
EFA: Exploratory Factor Analysis
CFA: Confirmatory Factor Analysis
ML: Maximum likelihood
CFI: Comparative-Factor Index
TLI: Tucker-Lewis Index
SRMR: Standardized root mean square residual
RMSEA: Root mean square error of approximation
CI: Confidence interval
